# Supplementary material for: Patterns of Intron Gain and Loss in Fungi
Source: PLoS Biol. 2004 Nov 30;2(12):e422. doi: 10.1371/journal.pbio.0020422 (PMC532390; doi:10.1371/journal.pbio.0020422)
Supplement: Table S1 — Also available at http://genes.mit.edu/NielsenEtAl/. (4.3 MB ZIP). [file pbio.0020422.st001.zip › NielsenEtAl/html/482.html]

AN0823.1.NCU06989.1.MG01456.1.FG05645.1


```
 CLUSTAL W (1.82) Multiple Sequence Alignments - Introns Inserted


Sequence 1: MG01456.1	529 aa
Sequence 2: FG05645.1	490 aa
Sequence 3: NCU06989.1	611 aa
Sequence 4: AN0823.1	504 aa
Alignment Length: 643 aa
Number Identitical Residues: 205 aa
Alignment Score (without introns) 10295


MG01456.1 	-----~-----------~------------------------------------------
NCU06989.1	MSGNL2GFFLFPRRGKR0IPRSCANHLGREAAHCPGADCVLSVIKEASKDDYVVSGPKTS
FG05645.1 	-----~-----------~------------------------------------------
AN0823.1  	-----~-----------~------------------------------------------
          	                                                            

MG01456.1 	-----------------------------------MAS--N0EPKLLYAIDGISAYHITN
NCU06989.1	FNKHLTKPVPCNGFQVRRNKYKAIRLPHHLQFHSGMASSHN0DPKLLYAINGVSAYHIAN
FG05645.1 	------------------------------------MS--N0DPKLLYAVEGINAYHITN
AN0823.1  	-----------------------------------MASTSN~DPRLLYSINNVHAFHMQN
          	                                     *: * :*:***:::.: *:*: *

MG01456.1 	GSEETLTPAGPQTLSLLMVPTSSPFADRSVDPK----SGKSEEDFYLHLHLPPELDLPLP
NCU06989.1	GKEESLTPAGPQTLSLLMVPTSSAFADPALG------TENAEQDFYLHLHLPPELDLPLP
FG05645.1 	GKEKSLTPSGPQTLSLLMVPTSSGFVEPAGN-------GSGEEDFYLHLHLPPELDLPLP
AN0823.1  	GEESSLTPSGPQTLSLLMVPTTTSIAENQNTAFSSAQETQPEEDFYLHLHLPPELDLALP
          	*.*.:***:************:: :.:    . :::.  . *:**************.**

MG01456.1 	ATTQIYHQPPRSYLIPRWDLGRDSGAFTRIEFPELGSRRH--IQEDVDTFETILAQCTAF
NCU06989.1	ATTQIYHQPPTSYLIPRWDLGPDSGAFTRIEFPSVESRKG--IQEDVDTFETILAQCTAF
FG05645.1 	ATTQIYHQPPTSYLIPRWDLGPDSGAFTRIEFPPANSRPG--VQEDVDTFETILAQCTAF
AN0823.1  	ATTQIYHQPPSSYLIPRWDLGPDAGAFIRIQFPGIGDGPNKVTQEDIDTFETILAQCTAF
          	********** ********** *:*** **:**   .   .  ***:*************

MG01456.1 	LERAPPPKLGVSSSSKGEKSGFGDEKAAARSMETGVAGPSSSKSKAALAQEPPPYNPNDF
NCU06989.1	LERAPPPRLNQGSNKSS------GEKGGTG---------TSTGSKGGVAEDLPPYNPGDY
FG05645.1 	LERSAPPHPPRSS-----------EKAQA-------------KAREAAGEQLPAYNPADY
AN0823.1  	HERAKVPN------------------------------------------DHASYNPASF
          	 **:  *.                                          : ..*** .:

MG01456.1 	QPGEAYARGSHSK------QQTGQIVLIDEEDGSVIGELGEGMTVIEHQALQPGSKE1PV
NCU06989.1	GPGEAYAQGSRSN------PTPGQIVLVDEDDGSVIGELAEGYQVHEDSALKPGSKD1PV
FG05645.1 	TPGENYAQGSQSA------NSAGRIVLIDEEDGSVLGELTDSYQVVEDKRIKPGSKE1PV
AN0823.1  	APGEGYVSSAGAAGPHGKTDPHGRIVLIDEENGSVVGEM-EGYDVVENPDVKPGSKR1PV
          	 *** *. .: : .. ..:   *:***:**::***:**: :.  * *.  ::****  **

MG01456.1 	EITLPTDSTQN-ISVAPASKESVEMLMHPAYQKSFLVSNASMASRFIVGTSDKVSSMLQS
NCU06989.1	EITLPTTPNGK-INVAPASPELFDAELHPAYKKSFLVSNASAASRFIITGSDMLAKLLQN
FG05645.1 	EIALPTDGGQN-ISVQPASQSWAEMDMHPAYKKSTLVSSASKASRLIVTTSDIVSRTLQS
AN0823.1  	QVQLPTEGEGNVVSVSNVSEEYLATARHPAYKNSSLVQSSATASRLIVTGSTYLANVMTS
          	:: ***    : :.*  .* .      ****::* **..:: ***:*:  *  ::  : .

MG01456.1 	GATAITAKSKPASKPMTFKPATHERIRRINTFTGGAVGLSTKTVGQIGKVAQNVGATLAR
NCU06989.1	QADNYTKRAQPAAEPMKFKPATREHIRRISHFTGNAAVLSAKTVGQIGKFAQNFGATLGG
FG05645.1 	QADNFTQKTKPNAKPVTFAPTTHEHIRRINTFSNRAATFSATTVGTIGNLAQNLGATVTR
AN0823.1  	GADAFTKKVKPNPKPATFSDATHTRIRKIGTFSSSAAEISSKTVGQVGRYAQNFGASLAS
          	 *   * : :* .:* .*  :*: :**:*. *:. *. :*:.*** :*. ***.**::  

MG01456.1 	KG-HKGGGKGFDKDGNPIDGYKPGLLNKSLMAFSTVADGIEQAGKTLLTSTSNAATTVVE
NCU06989.1	RGPRKTGTKGFDPDGQPLDNYKPGLLNKSFMAFSTVMDGVEQAGRHLLASTSEAATTVVA
FG05645.1 	R--KDGRARGYDKDGQAIDTYKPGVLNKSLMAFNTVVDGIEQAGRTLLNGTSSSVTTVVG
AN0823.1  	RK-DDGKARGDNSRVPPHG--KPGILNKSLIAFSTLADGIEQGARNVLTSGSTAASTMIG
          	:   .   :* :    . .  ***:****::**.*: **:**..: :* . * :.:*:: 

MG01456.1 	HKWGPEAGDVSRNLGSGVKNVGLVYIDVTGVSRRALVKNVAKGMIVGKAPNGKDVIVGGG
NCU06989.1	HKWGPEAGEISRSLGGGVKNVGLVYIDVTGVSRRAILKSVAKGMVVGRTADGGVVTVGGG
FG05645.1 	HRWGAEAGEVSRNLGGGVKNVGLVYIDVTGVSRRAILKSVAKGMVVGKVRGGGEIIVGGT
AN0823.1  	HRYGAEAGAVASNLTGGIKNVGLVYIDASGVSRKAVLKSVAKGMIVGRMKNGQQVVVGSG
          	*::*.*** :: .* .*:*********.:****:*::*.*****:**:  .*  : **. 

MG01456.1 	DGGVVPVPPEKRLKDSEGNGTSSSASSIKGYS-----DDKKAAIEYK
NCU06989.1	DGGVLSPGEGGMNSDPMNRAGRTSPGSIRGGKQPAGMNGRSETWG--
FG05645.1 	DNELIEA------RQGNGQGAQSTYGDNVSVA-----NGKKPA----
AN0823.1  	DGGDLSPPGPGLSPVGRRPSPSPTPPPAYGAPGTYSVGGASMSGGKR
          	*.  :              .  .:     .   . . .. . :
```
